# Contribution of T helper 17 cells and interleukin-17 to the pathogenesis of primary immune thrombocytopenia in Egyptian children

**DOI:** 10.1007/s00431-023-05242-3

**Published:** 2023-10-12

**Authors:** Tamer Hassan, Marwa Zakaria, Asmaa Diaa, Ayman E. L. Sayed Abdalla, AL Sayed M. Sayed Ahmed, Doaa M. Abdelmonem, Eman Abdelaziz

**Affiliations:** 1 Pediatrics, Department, Faculty of Medicine, Zagazig University, Zagazig, 44519 Egypt; 2 Ultra Medical Center, Alain-Abu Dhabi, United Arab Emirates; 3 AL-Dhafra Hospital, Seha, Abu Dhabi, United Arab Emirates; 4 Clinical Pathology Department, Faculty of Medicine, Zagazig University, Zagazig, Egypt

**Keywords:** Interleukin 17, Immune thrombocytopenia, ITP, Children

## Abstract

Though pathogenesis of primary immune thrombocytopenia (ITP) is still rendered unclear, yet there are many research efforts that have been directed to the role of T helper 17 (Th17) and interleukin 17 (IL-17) in the pathogenesis of this disease. The Th17 cell, which produces IL-17, is a subset of T helper cells. Interleukin 17 is pro-inflammatory cytokine that is recently proved to have a crucial role in the emergence of autoimmune diseases. We aimed to investigate the role of T helper17 cells and interleukin-17 in the pathogenesis of ITP in Egyptian children. This study was carried out on 100 children with ITP and 100 apparently healthy children as a control group. Patients were subjected to full medical history taking, thorough physical examination and routine investigations according to our local standards. Percentage of Th17 cells was measured by flow cytometry in study groups. Also, serum IL-17 was measured in in study groups by ELISA. Th 17 cells were significantly higher in patients compared to controls. Moreover, 3.1-fold increased serum levels of IL-17 were observed in patients with ITP compared to controls. Newly diagnosed patients had significantly higher percentage of Th-17cells as well as higher IL-17 levels than patients with either persistent or chronic ITP.

*Conclusion*: We concluded that Th 17 cells and IL-17 seem to play an important role in the pathogenesis of ITP in Egyptian children.

**Table:** 

**What is Known -- What is New:** *• The pathogenesis of ITP is heterogeneous A novel subset of CD4+ T cells, distinct from Th1 and Th2, was recently identified. It is characterized by the production of IL-17 and, therefore, designated as Th17 cells. Several studies support a pivotal role for serum cytokines in the pathogenesis of ITP and provide evidence to suggest that helper Tlymphocytes polarize into Th1 and Th2 immune response. we aimed to investigate the role of T helper17 cells and interleukin-17 in the pathogenesis of ITP in Egyptian children.*

**What is Known -- What is New:**

*• The pathogenesis of ITP is heterogeneous A novel subset of CD4+ T cells, distinct from Th1 and Th2, was recently identified. It is characterized by the production of IL-17 and, therefore, designated as Th17 cells. Several studies support a pivotal role for serum cytokines in the pathogenesis of ITP and provide evidence to suggest that helper Tlymphocytes polarize into Th1 and Th2 immune response. we aimed to investigate the role of T helper17 cells and interleukin-17 in the pathogenesis of ITP in Egyptian children.*

## Introduction

Primary ITP is the most common autoimmune cytopenia in childhood. It is characterized by isolated thrombocytopenia that is not accompanied by any other disorders that may lead to thrombocytopenia. Thrombocytopenia usually results from increased platelet destruction together with impaired platelet production. 2.2–5.3 per 100,000 children ≤ 18 years are diagnosed with primary ITP every year [1].

Despite major research efforts, the pathophysiology of ITP is not fully understood. Multiple mechanisms were proposed comprising antiplatelet antibodies secreted by auto reactive B lymphocytes, platelet destruction mediated by T cell, and poor functionality of regulatory T. cells accompanied with reduced number [2].

Innovative data had supported the hypothesis of CD4+ve T cell plasticity which in turn leads to diverse immune responses based on the type of impact made by the inflammatory environment; this could be exemplified in T. helper 17 cell [3].

Th17 cells are regarded as promoters of autoimmune conditions as they produce a number of pro-inflammatory cytokines including IL-17 and accordingly induce remarkable tissue damage [3].

IL-17 belongs to IL 17 cytokine family, which contain 6 different IL pro-inflammatory cytokines, from IL 17A to IL 17F. IL 17A is often presented as IL 17. The high degree of similarity between IL 17A and IL 17F is significant and they also have a common biological function [4].

IL-17 is a signature cytokine secreted from the Th 17 cells, apart from playing a crucial role in the pathogenesis of autoimmune diseases; it has shown to function differently from other members of IL 17 cytokine family. Interleukin 17 induces the expression of various cytokines and adhesion molecules [5].

IL-17 recruits and activates different cells to increase inflammation. Although protective in infection, over production of IL 17 promotes inflammation in autoimmune diseases such as multiple sclerosis, rheumatoid arthritis, and psoriasis [6].

Several studies demonstrated that genetic polymorphism of IL-17 was shown to be linked to various autoimmune diseases such as inflammatory bowel diseases, asthma, ITP, and psoriasis [7].

## Patients and methods

A case-control study was carried out in pediatric hematology outpatient clinic of Zagazig university hospitals during the period from March 2019 to April 2020. The study included 100 children with ITP who were recruited from pediatric hematology outpatient clinic and 100 age and sex matched apparently healthy children.

## Eligibility criteria

1. Patients with primary immune thrombocytopenia
2. Age from 1 to 15 years
3. Both sex

## Exclusion criteria

1. Patients with secondary immune thrombocytopenia
2. Patients with other causes of thrombocytopenia
3. Age < 1 year or > 15 years

## Diagnosis of ITP and classification

Diagnosis of ITP will be based on ASH clinical practice guidelines in 2011 as a platelet count less than 100,000/µL in the absence of other causes or disorders that may be associated with thrombocytopenia. ITP was classified as newly diagnosed (diagnosis to 3 months), persistent (3 to 12 months from diagnosis), or chronic (lasting for more than 12 months) [8].

## Study design: case-control study

### Methods

1. All patients were subjected to full history taking, thorough clinical examination and routine laboratory investigations for diagnosis and follow up of ITP according to our local standards including CBC using automatic cell counter (Sysmex XN2000, Japan) at diagnosis and during follow up as well as drawing platelet trend.
2. Measurement of Th-17cells was performed for all patients and controls by flow cytometry. Th-17cells were recognized by expression of intracellular IL-17. Briefly, heparinized peripheral blood (400 µL) with an equal volume of RPMI 1640 medium was incubated for 4 h at 37 °C, 5% CO_2_ in the presence of 25 ng/mL phorbol myristate acetate (PMA), 1 µg/mL ionomycin, and 1.7 µg/mL monensin (Alexis Biochemicals, San Diego, CA). After incubation, the cells were stained with PE-Cy5-conjugated anti-CD3 and FITC-conjugated anti-CD8 to delimitate CD4^+^ T cells because CD4 was down-modulated when cells were activated by PMA. After the surface staining, the cells were stained with PE-conjugated anti-IL-17A for Th17 detection after fixation and permeabilization. Stained cells were analyzed by flow cytometric analysis using BD FACS Calibur (BD Biosciences, USA). Th17 cells were identified as those that were CD3^+^CD8^−^IL-17A^+^.
3. Measurement of serum level of IL-17 was performed for all patients and controls using Enzyme Linked Immunosorbent Assay (ELISA) sandwich technique, using commercially available kit (Quantizing, R and D system, Inc. Minneapolis, USA) catalog number 201–12-0143 (Shanghai Sunred Biological Technology Co., Ltd). Briefly, the kit uses a double-antibody sandwich ELISA technique. A pre-coating with human IL-l7 monoclonal antibody was used to catch IL-17 and IL-l7 antibodies labeled with biotin, followed by streptavidin-HRP that was used for detection. A standard curve using five dilutions ranging from 37.5 to 600 pg/mL of IL17 was used for quantification. All samples were measured in duplicates. All tests were run simultaneously in one series.

## Statistical analysis

The data were checked, entered, and analyzed using SPSS version 20 (Armonk, NY: IBM Corp). Results were expressed as mean ± standard deviation for quantitative variables, and as number and percentage for qualitative ones. Unpaired Student’s *t*-test, chi-square test (*X*^2^), ANOVA (*F* test), and Pearson coefficient of correlation (*r*) were used when appropriate. *p* values ≤ 0.05 qualify as significant results and those ≤ 0.001 as highly significant results.

## Ethical approval

This study was conducted in accordance with the ethical standards of the Helsinki Declaration of 1964, as revised in 2000. The study protocol number (5126/15-1-2019) was approved by the Research Ethics Committee of the Faculty of Medicine, Zagazig University. Informed written consent and/or assent were obtained from the parents or guardians of each child.

## Results

The mean age of patients was 9.4 ± 3.5 years with a range from 3 to 15 years. The mean age at diagnosis was 6.9 ± 2.1. They were 56 males and 44 females. Based on ITP classification, 36 patients had newly diagnosed ITP, 30 patients had persistent ITP, and 34 patients had chronic ITP. Age at diagnosis was not significantly different among different groups of patients (*p* = 0.25). Patients with chronic ITP were significantly older than other patients (12.1, 9.3, and 7.0 for chronic, persistent and newly diagnosed ITP, respectively, *p* < 0.001). Though female gender was higher in chronic ITP compared to persistent and newly diagnosed patients, yet the difference did not reach a statistically significant level (53%, 33.3%, and 39%, respectively, *p* = 0.5).

The mean age of controls was 9.6 ± 3.5 with a range from 3 to 15 years. They were 50 females and 50 males. There was no significant difference between patients and controls as regards age and sex (*p* = 0.38 and *p* = 0.55, respectively).

Most of patients (92%) had purpura as the initial clinical presentation followed by ecchymosis in 86% of patients and external bleeding in 64% of patients. The mean initial platelet count was 12,000/µL. It was significantly higher in chronic ITP patients compared other patients (17,400, 13,200, and 6700/µL for chronic, persistent and newly diagnosed ITP, respectively, *p* < 0.001). Also, the mean initial platelet count was significantly higher in females compared to males (14,640/µL versus 10,200/µL, respectively, *p* = 0.03) (Table 1).

**Table 1:** Initial platelet counts in ITP patients based on disease classification and gender

	Platelets count (10^3^/µl) Mean ± SD (Range)	Test	*P*
• Newly diagnosed ITP	6.7 ± 4.2 (1–17)	*f* = 8.5	0.0007
• Persistent ITP	13.2 ± 7.8 (1–31)		
• Chronic ITP	17.4 ± 10.2 (2–33)		
• Males	10.2 ± 6.8 (1–31)	*t* = 1.8	0.03
• Females	14.64 ± 10.1 (2–33)		

*SD* standard deviation

Regarding the first line therapy, 14 patients were managed conservatively, 66 patients received steroids, 10 patients received intravenous immunoglobulin (IVIG), and 10 patients received combined steroids and IVIG. Regarding the second line therapy, 84 patients received thrombopoietin receptor agonists (TPO-RA), while 14 patients received azathioprine, and only 4% performed splenectomy.

Percentage of Th 17 cells was significantly higher in patients than controls (1.85% versus 0.82%, *p* < 0.0001). Newly diagnosed ITP patients had notably higher percentage of Th 17 cells in comparison to persistent and chronic ITP patients (Table 2).

**Table 2:** Percentage of Th-17 cells in our patients in relation to ITP classification

Th 17 cells	Newly diagnosed *N* = 36	Persistent *N* = 30	Chronic *N* = 34	Controls *N* = 100	*F* test	*P*
Mean ± SD (%)	2.05 ± 0.35	1.75 ± 0.21	1.53 ± 0.15	0.82 ± 0.11	36.7	< .00001
Range	1.74–2.35	1.55–2.15	1.25–1.88	0.71–0.95		

*Th-17* T helper 17, *SD* standard deviation

**Fig. 1:**
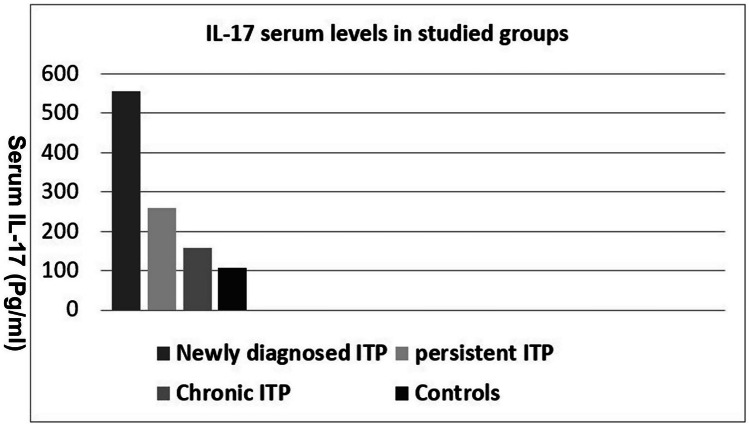
Shows the distribution of IL17 in the studied groups

Significantly higher levels of serum IL-17 were observed in patients compared to controls (331.4 pg/ml versus 106.7 pg/ml, respectively, *p* < 0.00001) (Fig. 1). Newly diagnosed ITP patients had notably higher serum IL-17 levels in comparison to persistent and chronic ITP patients (Table 3).

**Table 3:** Serum IL-17 levels in our patients in relation to ITP classification

Serum IL-17	Newly diagnosed ITP *N* = 36	Persistent ITP *N* = 30	Chronic ITP *N* = 34	Controls *N* = 100	*F* test	*P*
Mean ± SD (pg/ml)	554.3 ± 247.2	259.9 ± 209.0	158.6 ± 156.3	106.7 ± 76.6	17.0	< .00001
Range	163.2–949.5	65.0–764.8	63.2–580.2	63–341		

*IL-17* interleukin 17, *SD* standard deviation

There was no significant correlation between serum IL-17 levels and age at diagnosis (*r* =  − 0.03, *p* = 0.8) or platelet counts (*r* = 0.2, *p* = 0.1). Also, there was no significant association between serum IL-17 levels and any of clinical presentations (*p* = 0.07), first line therapy (*p* = 0.25), or second line therapy (*p* = 0.6).

## Discussion

The pathophysiology of ITP is extremely complex. Several studies support a pivotal role for serum cytokines in the pathogenesis of this disease and provide evidence to suggest that helper T-lymphocytes polarize into Th1 and Th2 immune response. Th1 response is characterized primarily by the presence of cytokines IL-2, INF-γ, and TNF-α, whereas Th2 response produces IL-4, IL-5, IL-6, IL-10, and IL-13 [2].

A novel subset of CD4+ T cells, distinct from Th1 and Th2, was recently identified. It is characterized by the production of IL-17 and, therefore, designated as Th17 cells. IL-17 is pro-inflammatory cytokine that recruits different cell types to the site of inflammation and thus play a protective role in infection. However, overproduction of IL-17 was reported in many autoimmune diseases [5].

In our study, percentage of Th 17 cells was significantly higher in patients than controls (1.85% versus 0.82%, *p* < 0.0001). Also, significantly higher levels of serum IL-17 were observed in patients compared to controls (331.4 pg/ml versus 106.7 pg/ml, respectively, *p* < 0.00001).

In 2009, Zhang and colleagues first described up-regulation of Th17 cells along with Th1 in patients with ITP and suggested that Th17 cytokines promoted an imbalance favoring a more Th1-type immune response in ITP [9].

Our results are also consistent with Ghallab et al. where there was a statistically significant difference between untreated ITP adult patients and controls as regards serum IL-17 levels (91.5 versus 59.9 pg/ml, respectively, *p* < 0.0001) [10].

Similarly, Ye et al. reported elevated Th17 cells as well as plasma IL-17and IL-23 levels in adult patients with ITP [11].

Zhou et al. investigated the role of interleukin-17-producing CD4-positive T cells in the pathogenesis of primary ITP and found that the percentage of Th17 and Th1 cells were markedly increased in ITP patients especially in those with severe ITP compared with normal controls. Further ELISA analysis verified high levels of Th17-associated pro-inflammatory cytokines such as interleukin-17A/F, interleukin-6, and interleukin-23 and low levels of inflammatory inhibitory factors including interleukin-10 and transforming growth factor-β in ITP patients compared with normal controls [12].

On the contrary, Ma et al. found that plasma IL-17 levels were not significantly different between adult patients with active ITP (median, 15.04 pg/ml (range, 8.15–66.78)) and the control group (median, 15.27 pg/ml (range, 10.25–40.36); *p* = 0.17) [13]. Also, there was no significant difference of the other Th17 associated cytokines (TGF-ß and IL-6) and Th1 cytokine (IFN-γ) were observed between ITP patients and controls. This discrepancy can be attributed to the difference in the study population and sample size where Ma et al. study was carried out on only 29 adults with ITP where our study was carried out on 100 children with ITP.

Our results showed that newly diagnosed ITP patients had notably higher percentage of Th 17 cells and IL-17 serum levels in comparison to persistent and chronic ITP patients (2.05, 1.75, and 1.53%, respectively, *p* < 0.0001 for T helper cells and 554.3, 259.9, and 158.6 pg/ml, respectively, *p* < 0.001 for IL-17).

Our results are in agreement with Huang et al. where they found that the levels of IL-17 were lower in patients with chronic ITP than those with newly diagnosed ITP and comparable to the control group [14].

Ghallab et al. found the level of IL-17 was increased in adult patients with untreated ITP (*p* = 0.0001) when compared with controls. However, there was statistically significant reduction in the level of IL-17 in responder patients (*p* = 0.0001) while IL-17 level was insignificantly changed in non-responder patients (*p* = 0.394) [10].

Zhang and colleagues found that among the ITP patients, there were no statistical differences of the three kinds of cells (Th 17, Th1, and Tc1) tested between primary and recurrent ITP patients (*p* = 0.18 for Th17, *p* = 0.36 for Th1, *p* = 0.35 for Tc1) [9].

Ye et al. did not observe any statistical difference in plasma IL-17 levels between adults with newly diagnosed ITP and those with recurrent ITP [11].

El Husseiny et al. in their study over 45 adult patients with chronic and persistent ITP found significantly higher levels of IL-17 in their patients compared to control group (0.42 versus 0.15, *p* < 0.001) [15].

Our results can be explained based on the clinical differences between newly diagnosed and chronic ITP which suggest the existence of different pathophysiological mechanisms in the two forms [2].

In our study, there was no significant correlation between serum IL-17 levels and age at diagnosis (*r* =  − 0.03, *p* = 0.8) or platelet counts (*r* = 0.2, *p* = 0.1). Also, there was no significant association between serum IL-17 levels and any of clinical presentations (*p* = 0.07), first line therapy (*p* = 0.25), or second line therapy (*p* = 0.6).

Very few studies investigated the relationship between serum IL-17 levels and demographic, clinical or laboratory parameters in childhood ITP.

Ma et al. found no significant association between plasma IL-17 levels and any of age, sex, or platelet counts [13].

El Husseiny et al. observed insignificant correlation between IL-17 levels and platelet counts [15].

Okamoto et al. classified their patients into the IL-17-low-expression group and IL-17-high-expression. The clinical information between the IL-17-low-expression group and IL-17-high-expression group did not significantly differ for age, sex, and platelet counts [16].

## Conclusion

We concluded that Th 17 cells and IL-17 seem to play an important role in the pathogenesis of ITP in Egyptian children. Larger multicenter studies are still needed to support our findings.

## Limitation of the study

Small sample size was one of the limitations in this study and so larger multicenter studies are still needed to support these findings. Another limitation was that we need to start with patients with de novo ITP and to follow the changes in percentage of T helper 17 cells and serum levels of IL-17 over time. However, many patients with de novo ITP lost follow up especially after improvement.

## Data Availability

The data sets generated during and/or analyzed during the current study are available from the corresponding author on reasonable request.

## Abbreviations

IL: Interleukin
ITP: Immune thrombocytopenia
Th: T helper
ELISA: Enzyme Linked Immunosorbent Assay
ASH: American society of hematology
INF: Interferon
TNF: Tumor necrosis factor
TPO-RA: Thrombopoietin receptor agonists
TGF: Transforming growth factor
ANOVA: Analysis of variance
